# Tinea pedis and onychomycosis frequency in diabetes mellitus patients and diabetic foot ulcers. A cross sectional – observational study

**DOI:** 10.12669/pjms.324.10027

**Published:** 2016

**Authors:** Gamze Akkus, Mehtap Evran, Dilek Gungor, Mehmet Karakas, Murat Sert, Tamer Tetiker

**Affiliations:** 1 Dr. Gamze Akkus, M.D. Specialist in Internal Medicine, Department of Internal Medicine, Division of Endocrinology, Cukurova University Medical Faculty, 01330 Adana, Turkey; 2 Dr. Mehtap Evran, M.D. Specialist in Internal Medicine and Endocrinology, Department of Internal Medicine, Division of Endocrinology, Cukurova University Medical Faculty, 01330 Adana, Turkey; 3 Dr. Dilek Gungor, M.D. Research Assistant in Dermatology, Department of Dermatology, Department of Internal Medicine, Division of Endocrinology, Cukurova University Medical Faculty, 01330 Adana, Turkey; 4 Prof. Dr. Mehmet Karakas, M.D. Specialist in Dermatology, Department of Dermatology, Cukurova University Medical Faculty, 01330 Adana, Turkey; 5 Prof. Dr. Murat Sert, M.D. Specialist in Internal Medicine and Endocrinology, Department of Internal Medicine, Division of Endocrinology, Cukurova University Medical Faculty, 01330 Adana, Turkey; 6\ Prof. Dr. Tamer Tetiker, M.D. Specialist in Internal Medicine and Endocrinology, Department of Internal Medicine, Division of Endocrinology, Cukurova University Medical Faculty, 01330 Adana, Turkey

**Keywords:** Diabetic foot ulcer, Diabetes mellitus, Onychomycosis, Tinea pedis

## Abstract

**Objective:**

Impaired cellular immunity and reduced phagocytic function of polymorphonuclear leukocytes facilitate the development of skin fungal and bacterial infections due to uncontrolled hyperglycemia in diabetic patients. In our study, we aimed to assess onychomycosis and/or tinea pedis frequency in diabetic patients, and effects on the development of chronic complications, particularly foot ulcer.

**Methods:**

We included 227 diabetic patients in the study. Forty-three patients had diabetic foot ulcer. We screened and recorded demographic characteristics, HbA1c levels of patients, and presence of complications We examined patients dermatologically, and collected samples by scalpel from skin between toes, and from sole, toe nail, and area surrounding nails from suspected to have fungal infection.

**Results:**

Native positivity between toes was higher in men compared to women (p<0.05). We obtained significant relation between HbA1c elevation and native positivity between toes (p<0.05). Fungal infection between toes, at sole and toe nail significantly increased in patients with diabetic foot ulcer compared to patients without diabetic foot ulcer (p<0.05). Moreover, native positivity in patients with diabetic foot ulcer correlated with presence of fungal infection examination findings (p<0.05).

**Conclusion:**

Fungal infections were more frequently observed in the presence of poor glycemic control and peripheral vascular disease in diabetic patients in compliance with the literature, and the presence of fungal infection may also responsible for the development of foot ulcers.

## INTRODUCTION

Diabetes mellitus is one of the significant chronic and metabolic disease with gradually increasing frequency.^1^,^2^ Various skin lesions are observed in approximately 30% of diabetic patients, and fungal skin infection are significant part of these lesions.^3^ It is considered that chronic hyperglycemia in patients affects cellular immunity and polymorphonuclear leukocytes, and impairs phagocytic functions. As a result of this condition, development of cutaneous fungal and other bacterial infections are seen in these patients.^4^,^5^

Although tinea infections are minimally symptomatic in non-diabetic patients, they may form entry points and fistulas which lead to serious bacterial infections in diabetic patients. Involved areas are pruritic, squamous, erythematous, and macerated. Vesicles and pustules may be formed. Long lasting tinea pedis may also involve the nails by forming thick, yellowish brown, and rough nail surface, and subungual debris.^6^

In this study, we aimed to assess onychomycosis and/or tinea pedis frequency in diabetic patients, and effects on the development of chronic complications, particularly foot ulcer.

## METHODS

We included 227 patients, followed up with diabetes diagnosis in 2014 at Endocrinology Outpatient Clinic and/or Inpatient Clinic in the present study. Forty-three of these patients had diabetic foot ulcer. Demographic information of all patients was recorded on a questionnaire form by a face to face interview with the patient’s consent. Information related to age, gender, type and duration of diabetes, hemoglobinA1c (HbA1c) levels, and daily feet washing frequency were recorded in this questionnaire. We also recorded informations related to neuropathy and peripheral vascular system diseases, which are chronic complications of diabetes by reviewing the available patient records. Lesions in patients with diabetic foot ulcer were assessed according to Wagner classification.

All patients had a full dermatological examination. Skin at sole and between toes, nail, and area around nail were cleaned with alcohol 70%, and samples were collected by using scalpel. Materials were collected from the skin between 4^th^ and 5^th^ toes of normal appearance areas, and intense squamous hyperkeratotic, macerated or squamous area from toes and foot sole. Materials were collected in sterile paper envelopes. Samples were collected from the deepest section of distal nail plate from normal appearance, and from the most affected section of the nail in cases suggesting onychomycosis. Lesions were examined at microscope by X40 magnification, and in case non-living, densely branched and sectioned hyphae with hyaline hypha and/or arthroconidia chains were observed, they were assessed as “positive fungal test in direct microscopic examination”. In case of negative fungal test, this procedure was repeated twice at the same area.

Statistical analysis of data was performed by using SPSS program. Descriptive assessments and frequency analysis were performed. P<0.05 was accepted as statistically significant.

## RESULTS

Total 141 patients (62.1%) were female, and 86 patients (37.9%) were male (Table-I). The most frequently identified fungal infection in our patients was onychomycosis (34.9%), and the second was tinea pedis (26.3%). About 22.7% of female patients and 45.3% of male patients were natively positive. While native positivity between toes and at sole was more observed in male patients (p<0.05, p<0.05; respectively), native positivity at toe-nail was detected as similar in both genders (p>0.05). Native positivity between toes and at sole increased as correlated with increasing age, and native positivity between toes increased as correlated with increasing mean HbA1c levels (p<0.05, p<0.05; respectively). No relationship was detected between mean duration of diabetes, professions, treatment method, presence of co-morbid diseases, and native positivity. Incidence of fungal infection was found to be widespread at skin between toes, at sole, and at toe nail in patients with peripheral vascular diseases (p<0.05). Between the presence of peripheral neuropathy and incidence of tinea pedis we did not detect any relationship. Mean frequency of daily foot washing was 2.7±2.0/day in patients participating in the study, and foot washing frequency was significantly higher in women compared to men (p=0.0001). Meanwhile when frequency of foot washing decreased, fungal infection increased at sole and toe nail (p=0.004). However, there was not any significant relation between frequency of foot washing and type of treatment received by patients, presence of diabetic foot ulcer, peripheral vascular disease, and peripheral neuropathy.

**Table-I:** Demographic and clinical characteristics of patients.

	Number of Patients (n, %)	Mean ± SEM
Age (years) (Female)		54.8 ± 12.8
(Male)		56.9 ± 11.9
HbA1c (%)		8.28+2.21
Duration of diabetes (years)		10.5 ± 6.9
Type 1 DM*	10, 1.9%	
Type 2 DM*	227, 98.1%	
OAD drug use**	81, 35.7%	
OAD + Insulin use**	146, 64.3%	
Foot ulcer presence	43, 18.9%	
Vascular disease presence***	52, 23%	
Neuropathy	71, 33.6%	

* DM: Diabetes mellitus,

** OAD: Oral antidiabetic

*** Vascular disease presence has been detected by lower extremity doppler ultrasonography.

Fungal infection between toes, at sole, and at toe nail was found significantly higher in patients with diabetic foot ulcer compared to patients without foot ulcer (p<0.05, p<0.05, p<0.05; respectively). In patients with diabetic foot ulcer, native positivity correlated with the presence of fungal infection as per examination findings (p<0.05). Fungal infection examination findings and native results of diabetic foot ulcers are shown in Table-II.

**Table-II:** Between toes, sole, and toe nail native results of patients with no diabetic foot ulcer and patients with diabetic foot ulcer.

	Total (n, %)	Native (+)	*p value
Between toes	227	71, 31.3%	0.001
Patients with no diabetic foot ulcer			
Between toes	184	46, 25%	0.000
Patients with (+) examination finding**			
Sole	164	48, 29.3%	0.005
Toe nail	109	38, 34.9%	0.22
Patients with diabetic foot ulcer***			
Between toes	43	25, 58.1%	<0.05
Patients with (+) examination finding**			
Sole	41	22, 53.7%	<0.05
Toe nail	27	16, 59.3%	<0.05

* p<0.05: accepted as significant

** Fungal infection skin findings: Erythematous - squamous lesion, hyperkeratosis, desquamation and/or vesicles at sole; maceration, squama, and/or fissures between toes; discoloration, onycholysis, subungual hyperkeratosis, and nail dystrophy at toe nails

*** Diabetic foot ulcers have been assessed according to Wagner classification.

## DISCUSSION

We observed fungal infections more frequently in the presence of poor glycemic control and peripheral vascular disease in diabetic patients. In addition more frequent fungal infection was seen in the presence of foot ulcers.

Local or systemic infections are more frequently observed in diabetic patients compared to normal population, and generally have a more serious course.^7^ A significant part of these infections consists of onychomycosis and tinea pedis.^8^ Even though it is harmless in many patients, tinea pedis may form entry points and fistula which will lead to serious bacterial infections in diabetic patients.^5^

In our patients, the most frequent fungal infection was onychomycosis (34.9%), followed by tinea pedis (26.3%). The results of similar studies performed in different regions of Turkey also support our findings.^9^,^10^ A non-controlled study including 288 diabetic cases, reported the prevalence of onychomycosis as 22%. Onychomycosis prevalence was reported as 4.9% in Western European population at similar age group, and it has been reported that diabetes is a metabolic disease facilitating onychomycosis.^11^,^12^,^13^ Various factors facilitating the formation of onychomycosis (peripheral vascular disease, immunosuppression, trauma, and peripheral neuropathy et al.) are more frequently observed in diabetic patients compared to normal population,^14^,^15^ Similarly in our study, a significant relation has been detected between presence of peripheral vascular disease and onychomycosis.

In our study, number of patients with peripheral neuropathy was found lower compared to literature,^16^ and no significant relationship was detected between presence of neuropathy and onychomycosis. Gender may be a determinant in onychomycosis formation by the presence of peripheral neuropathy.^17^ Onychomycosis is more frequently observed in men compared to women in general population, perhaps the reason of more activity in social life, their nails are more exposed to trauma, and they use close-toe shoes more frequently.^18^ Some studies in diabetic patients as in our study, suggest that onychomycosis frequency is more often in men, Ilkit et al. demonstrated equal rates in both genders.^19^ Shahzad et al. have found fungal infections associated with diabetes duration.^20^

In non-controlled or non-diagnosed diabetics, incidence and morbidity of fungal infections may be reduced by control of diabetes, effective diabetes regulation time and use of antifungal agents^20^,^21^ It has been suggested that the incidence of other superficial fungal infections such as onychomycosis and tinea pedis would be lower in patients with normal HbA1c levels. So that the study conducted by Alteras et al. supporte this opinion,^22^ Romano et al. reported that no relationship was present between dermatophytes infections and diabetes duration, HbA1c, and glucose levels.^23^

Unlike in our study, elevated HbA1c levels accompanied by high incidence of tinea pedis. However, we think that we may reach to more reliable results on this subject by studies with longer follow up periods for HbA1c levels.

Most studies related with fungal infections in diabetic patients focus on onychomycosis.^24^,^25^ Factors facilitating onychomycosis such as peripheral vascular disease, immunosuppression, trauma, peripheral neuropathy, and age are also applicable for tinea pedis.^25^,^26^ In our study, tinea pedis has been detected as the second most frequent fungal infection following onychomycosis in diabetic patients. Again in our study, it has been shown that age of diabetic patients, and complications such as peripheral circulatory failure and diabetic foot ulcer were factors facilitating onychomycosis and tinea pedis.

Since development of diabetic foot ulcer may lead to serious complications such as development of cellulitis, osteomyelitis, thrombophlebitis infection, foot infections are quite important in diabetic patients.^21^ Chronic erosions and ulcers may lead to an entry for bacterial and fungal infections, and afterwards increased morbidity and lower extremity amputations with delayed wound healing. One of the important factor affecting the frequency of onychomycosis or tinea pedis in diabetic patients is insufficient foot care training to patients, and their varying abilities to apply this training.^27^ In our patients as well, the frequency of feet washing was assessed, and it has been demonstrated that as frequency of feet washing decreased, presence of fungal infection increased.

We also observed more native positivity in patients with fungal infection examination finding. And this indicates the importance of physician experience in terms of detecting fungal infection in diabetic patients.

### Limitations of the study

One of the most significant limitations of our study is the absence of comparison of fungal infections of diabetic patients with a healthy control group. Another limitation of our study is the low number of patients with diabetic foot ulcer.

We found that fungal infections may be observed more frequently in the presence of poor glycemic control and peripheral circulatory failure in diabetic patients. Moreover, it has been suggested that fungal infections may contribute to development of foot ulcer in diabetic patients, since fungal infections are more frequently observed in diabetic patients with foot ulcer. Training of diabetic patients on infections, complications, the prevention, and ensuring early treatment are the cornerstones of the above mentioned disease.
